# Endotoxin-induced myocardial dysfunction in senescent rats

**DOI:** 10.1186/cc5033

**Published:** 2006-08-30

**Authors:** Sandrine Rozenberg, Sophie Besse, Hélène Brisson, Elsa Jozefowicz, Abdelmejid Kandoussi, Alexandre Mebazaa, Bruno Riou, Benoît Vallet, Benoît Tavernier

**Affiliations:** 1 Université Lille 2, Laboratoire de pharmacologie, EA 1046, Centre hospitalier universitaire (CHU) de Lille, Lille, France; 2 Fédération d'anesthésie réanimation, CHU de Lille, Lille, France; 3 Laboratoire de recherche sur la croissance cellulaire, la réparation et la régénération tissulaires, UMR CNRS 7149, Université Paris 12 – Val de Marne, Créteil and Université René Descartes – Paris 5, Paris, France; 4 INSERM-Institut Pasteur, U545, 1 rue du Pr. Calmette, Lille, France; 5 Université Denis Diderot – Paris 7, Laboratoire d'anesthésiologie, EA 322, Département d'anesthésie-réanimation, CHU Lariboisière, Assistance Publique-Hôpitaux de Paris (AP-HP), Paris, France; 6 Université Pierre et Marie Curie – Paris 6, Laboratoire d'anesthésiologie, EA 3975, Service d'accueil des urgences, CHU Pitié-Salpêtrière, Assistance Publique-Hôpitaux de Paris, Paris, France

## Abstract

**Introduction:**

Aging is associated with a decline in cardiac contractility and altered immune function. The aim of this study was to determine whether aging alters endotoxin-induced myocardial dysfunction.

**Methods:**

Senescent (24 month) and young adult (3 month) male Wistar rats were treated with intravenous lipopolysaccharide (LPS) (0.5 mg/kg (senescent and young rats) or 5 mg/kg (young rats only)), or saline (senescent and young control groups). Twelve hours after injection, cardiac contractility (isolated perfused hearts), myofilament Ca^2+ ^sensitivity (skinned fibers), left ventricular nitric oxide end-oxidation products (NOx and NO_2_) and markers of oxidative stress (thiobarbituric acid reactive species (TBARS) and antioxidant enzymes) were investigated.

**Results:**

LPS (0.5 mg/kg) administration resulted in decreased contractility in senescent rats (left ventricular developed pressure (LVDP), 25 ± 4 vs 53 ± 4 mmHg/g heart weight in control; *P *< 0.05) of amplitude similar to that in young rats with LPS 5 mg/kg (LVDP, 48 ± 7 vs 100 ± 7 mmHg/g heart weight in control; *P *< 0.05). In contrast to young LPS rats (0.5 and 5 mg/kg LPS), myofilament Ca^2+ ^sensitivity was unaltered in senescent LPS hearts. Myocardial NOx and NO_2 _were increased in a similar fashion by LPS in young (both LPS doses) and senescent rats. TBARS and antioxidant enzyme activities were unaltered by sepsis whatever the age of animals.

**Conclusion:**

Low dose of LPS induced a severe myocardial dysfunction in senescent rats. Ca^2+ ^myofilament responsiveness, which is typically reduced in myocardium of young adult septic rats, however, was unaltered in senescent rats. If these results are confirmed in *in vivo *conditions, they may provide a cellular explanation for the divergent reports on ventricular diastolic function in septic shock. In addition, Ca^2+^-sensitizing agents may not be as effective in aged subjects as in younger subjects.

## Introduction

Impairment in cardiac function is one of the most recognized organ dysfunctions in sepsis. Although the mechanism of myocardial dysfunction is complex and remains incompletely defined, increasing experimental evidence suggests that the main subcellular mechanisms include decreased cardiac myofilament responsiveness, nitric oxide (NO)-peroxynitrite activation, and inhibition of mitochondrial oxidative phosphorylation [1]. Surprisingly, while sepsis predominantly affects older persons, and although this segment of population will increase significantly in intensive care units over the coming years, only few experimental data on septic organ dysfunction in the aged animal are available.

A senescent heart is characterized by a progressive decline in contractile function, with slowing of twitch contraction, altered Ca^2+ ^handling kinetics, and impaired β-adrenergic modulation of contractility [2-4]. Aging is also characterized by an altered immune function and response to stress, including endotoxic challenge [5]. Septic myocardial dysfunction may thus be altered with aging in its severity, mediators, and/or main cellular mechanisms.

We have previously shown in young adult animal models of endotoxemia that troponin I phosphorylation decreases myofilament Ca^2+ ^sensitivity and may contribute to the depression of cardiac contractility [6,7]. The role of this alteration in the pathophysiology of septic myocardial depression has recently been confirmed in transgenic mice with cardiac-specific expression of slow skeletal troponin I (which lacks the protein kinase A phosphorylation sites) [8]. Reduced myofilament Ca^2+ ^sensitivity is also proposed as a cellular basis for the ventricular dilation described in fluid-resuscitated septic patients [9]. Therapeutic implications have recently been shown with the beneficial use of levosimendan, a new 'Ca^2+^-sensitizing' agent, in both endotoxemic animals [10] and septic shock patients with left ventricular dysfunction [11].

Whether these experimental findings and their clinical implications are relevant to septic dysfunction in the senescent heart is not known. To test this hypothesis, we established a model of myocardial dysfunction in senescent endotoxemic rats derived from a model of myocardial dysfunction during mild endotoxemia in the young adult rat [7,12], and we assessed cardiac contractility and myofilament Ca^2+ ^responsiveness in both young adult rats and senescent rats. In order to characterize more precisely the impact of aging on septic cardiac dysfunction, we also investigated several of its putative mediators (NO pathway and oxidative stress) in hearts from endotoxemic animals.

## Materials and methods

### Animal models

All procedures conformed to the framework of the French legislation that controls animal experimentation. Experiments were carried out in young (3 months old) and senescent (24 months old) male Wistar rats (Charles River Laboratories, L'Arbresle, France). Twenty-four months old is the age in Wistar rats at which natural mortality (since birth) is 50%, a rate that defines senescence.

Young adult rats were given an intravenous injection of lipopolysaccharide (LPS) endotoxin (*Escherichia coli *0111:B4 from a single batch (number 31K4121), 5 mg/kg; Sigma-Aldrich, Saint Quentin Fallavier, France) or of saline in the dorsal penile vein under brief halothane anesthesia. Animals were thereafter conscious and unresuscitated until the time of killing 12 hours later. Following LPS injection, animals exhibited prostration and moderate body weight loss. Mortality 12 hours after LPS injection was less than 10%, as previously reported [7,12].

In preliminary experiments, doses of LPS used in young adults (5 mg/kg) induced 100% mortality in senescent rats within the first 12 hours of endotoxemia. Mortality at 12 hours was still greater than 50% in senescent rats injected with doses of 1–2 mg/kg. In contrast, the injection of 0.5 mg/kg LPS induced a model of endotoxemic senescent rats where external appearance, weight loss, and mortality were very similar to that observed in young rats receiving 5 mg/kg, and this smaller dose was thus chosen for the study.

To allow interpretation of the results, another group of young adult rats received a dose of 0.5 mg/kg. In summary, five groups of animals were thus studied: two groups of senescent rats (control or LPS 0.5 mg/kg) and three groups of young adult rats (control, LPS 0.5 mg/kg, or LPS 5 mg/kg). The same volume was injected in all groups.

### Isolated Langendorff-perfused heart

Twelve hours after LPS or saline injection, the rats were anesthetized with an intraperitoneal injection of thiopenthal sodium (Nesdonal, Specia; Rhône-Poulenc, Paris, France). The hearts were then rapidly excised and perfused according to the Langendorff method at a perfusion pressure of 75 mmHg. The perfusate was a Krebs–Henseleit solution containing NaCl 118 mmol/l, NaHCO_3 _25 mmol/l, KCl 4.75 mmol/l, KH_2_PO_4 _1.18 mmol/l, MgSO_4 _1.17 mmol/l, CaCl_2 _1.25 mmol/l, glucose 10 mmol/l (pH 7.4, 37°C), and was bubbled constantly with 95% O_2_/5% CO_2_, as previously reported [7].

The left ventricular pressure was measured using a compliant water-filled balloon, connected to a pressure transducer (SensoNor SP 844; Capto, Horten, Norway) via a rigid polyethylene tube introduced into the left ventricle through the mitral valve, and was recorded on a Power Lab acquisition system (ADInstruments Pty Ltd, Castle Hill, Australia). The hearts were paced at 300 beats/minute via electrodes placed on the left atrial wall and connected to a stimulator (6002 model; Harvard Biosciences, Les Ulis, France). The collapsed balloon was filled with saline to obtain a left ventricular end diastolic pressure of 5 mmHg. After 15–20 minutes of equilibration, the left ventricular developed pressure (LVDP), the peak of the positive and negative pressure derivatives (respectively, d*P*/d*t*_max _and -d*P*/d*t*_max_) as well as the coronary flow were recorded.

### Skinned myocardial fibers

After excision of the heart, ventricular fiber bundles (approximately 200 μm in diameter) were dissected from left ventricular papillary muscles in a relaxing solution free of Ca^2+ ^(see composition below). Fibers were then incubated for one hour in a relaxing solution containing 1% Triton X-100 to solubilize the membranes without affecting the contractile proteins, as previously reported [13]. After the skinning procedure, one bundle was mounted between a fixed end and a force transducer (FT-03C model; Grass Instruments, Quincy, MA, USA) in a 0.8 ml chamber filled with the relaxing solution, adjusted to slack length, stretched by 20%, and subjected to an activation/relaxation cycle. The muscle contraction was amplified on a differential amplifier (Biological Amplifier 120; BioScience, Washington, DC, USA) connected to a recorder (TA240 model; Gould Electronic, Cleveland, OH, USA). The length and diameter of the muscles were measured by use of a graticule in the dissecting microscope. The sarcomere length in our setup was verified by a calibrated micrometer for several bundles from adult and senescent rats under a 400× Zeiss lens. Values ranged between 2.2 and 2.4 μm for all bundles tested. For all the following described experiments, the length of the fibers was kept constant to avoid sarcomere length-dependent changes in Ca^2+ ^sensitivity. All experiments were performed at constant temperature (22°C).

The relaxing solution contained 3-(*N*-morpholino)-propane sulfonic acid 10 mmol/l, potassium propionate 170 mmol/l, magnesium acetate 2.5 mmol/l, and K_2_-EGTA 5 mmol/l. Activating solutions had the same composition as the relaxing solution except that Ca^2+^-EGTA was substituted for K_2_-EGTA at various ratios. The concentrations of the different components in the solutions were calculated using program 3 of Fabiato and Fabiato [14] to keep the ionic strength at 200 mM. Solution also contained ATP (2.5 mmol/l) and phosphocreatine (10 mmol/l), and the pH was 7.00 ± 0.01. Free Ca^2+ ^concentrations of activating solutions ranged from pCa 6.2 ([Ca^2+^] = 0.63 μM) to pCa 4.6 ([Ca^2+^] = 25.0 μM, maximally activating solution), where pCa = -log_10_[Ca^2+^]. All chemicals were obtained from Sigma-Aldrich.

For each skinned cardiac bundle, the resting tension was first recorded. Measurements of active developed tension were then performed after stepwise exposure of the fibers from pCa 6.2 to pCa 4.6. To quantify myofilament Ca^2+ ^sensitivity, intermediate tensions were expressed as a percentage of the maximal tension obtained at pCa 4.6. Data were fitted using a nonlinear fit of the Hill equation (EnzFitter 1.05; Biosoft, Cambridge, UK). The slope coefficient (Hill coefficient) as well as the pCa value for half-maximal tension (pCa_50_) were calculated for each bundle.

### Myocardial nitric oxide content

The NO content in the left ventricle was determined as the NO end-oxidation products (nitrate and nitrite) (NOx). The NOx measurements were performed using the Griess reaction as previously reported [15]. Briefly, the nitrate present in samples was stoichiometrically reduced to nitrite in the presence of reduced nicotinamide adenine dinucleotide and nitrate reductase, for 15 minutes at 37°C. Total nitrite was mixed with sulfanilamide and *N*-(1-naphtyl)ethylenediamine dihydrochloride at room temperature for 10 minutes to generate a red–violet diazo dye that was measured on the basis of its absorbance at 550 nm. The nitrite concentration was determined from a standard curve generated using potassium nitrite. Results were normalized for protein concentration.

### Lipid peroxidation

The level of lipid peroxidation, a marker of oxidative injury, was assessed via thiobarbituric acid reactive substances (TBARS) formation during an acid-heating reaction, as previously described [16]. Briefly, left ventricular tissue was homogenized in phosphate buffer at 4°C. A 100 μl homogenate was pipetted into a test tube, followed by addition of 100 μl of 8.1% sodium dodecylsulfate, 750 μl of 0.8% thiobarbituric acid, 750 μl of 20% acetic acid (pH 3.5), and the volume was made up to 2.0 ml with distilled water. To minimize peroxidation during the assay procedure, 50 μl of 0.8% butyl-hydroxytoluene solution in acetic acid was added to the thiobarbituric acid reagent mixture. Tube contents were then boiled at 95°C for one hour. Following cooling to room temperature and centrifugation (4,000 rpm for 10 minutes), the absorbance of the supernatant was read spectrophotometrically at 532 nm. The amount of TBARS was calculated from a standard curve using malondialdehyde as a standard. Results were expressed in malondialdehyde equivalents per milligram of protein.

### Antioxidant enzyme activities

Superoxide dismutase (SOD), catalase (CAT), and glutathione peroxidase (GPX) activities were measured in myocardial tissue as previously reported [17,18]. Briefly, the SOD activity was assayed by measuring the inhibition of oxidation of 2-(4-idiophenyl)-3-(4-nitrophenol)-5-phenyltetrazolium to yield a chromophore with maximal absorbance at 505 nm, using a commercially available kit (Ransod; Randox, San Diego, CA, USA). The CAT activity was determined after sonication of the tested sample in phosphate buffer, as the rate of decrease in hydrogen peroxide absorbance at 240 nm, according to Aebi [17]. The GPX activity was measured as described previously [18] using a colorimetric assay kit (Cellular GPX Assay kit; Calbiochem, San Diego, CA, USA), where GPX activity is derived from quantification of NADPH oxidation (absorbance at 340 nm) in a solution also containing reduced glutathione, glutathione reductase, and *tert*-butyl hydroperoxide to initiate the reaction. All results were normalized for protein concentration.

### Statistical analysis

All values are expressed as the mean ± standard error of the mean. Comparisons between control and LPS groups were made using an unpaired Student's *t *test or, when more than two groups were compared, an analysis of variance and the Fisher's PLSD *post hoc *test. Where needed, the interaction between age and LPS was tested using a two-way analysis of variance. All *P *values were two-tailed, and *P *< 0.05 was required to reject the null hypothesis. Statistical analysis was performed with Statview 5.0 software (SAS Institute Inc., Cary, NC, USA).

## Results

Changes in the body weight and main organ weight in response to 12-hour endotoxemia in each age group are summarized in Table 1. In senescent rats, LPS induced a body weight loss and an increase in heart weight and lung weight similar to those observed in both groups of young rats treated with LPS. In addition, a significant liver weight loss was present in young rats after LPS 5 mg/kg only, and was present in senescent rats (Table 1). As expected from preliminary experiments and previous studies, mortality was lower than 10% in all LPS groups.

### Isolated Langendorff-perfused hearts

LPS 5 mg/kg in young rats induced a contractile dysfunction, as reported by an approximately 50% decrease of LVDP and other indices of myocardial function (Table 2). The reduction in LVDP observed in young rats injected with 0.5 mg/kg suggested that depression of contractility may be dose dependent, although the difference between the two LPS-injected young groups did not reach statistical significance (Table 2). In senescent hearts, left ventricular function was altered in basal conditions. A marked depression, of a relative amplitude similar to that recorded in young rats after LPS 5 mg/kg, was observed in senescent rats after LPS 0.5 mg/kg (Table 2). Two-way analysis, however, showed no significant interaction between age and LPS 0.5 mg/kg. The effect of LPS 0.5 mg/kg on the LVDP therefore did not differ according to age. Coronary flow was not altered by endotoxemia, whatever the age of the rats (Table 2).

### Myofilament Ca^2+ ^responsiveness in skinned fibers

**Figure 1:**
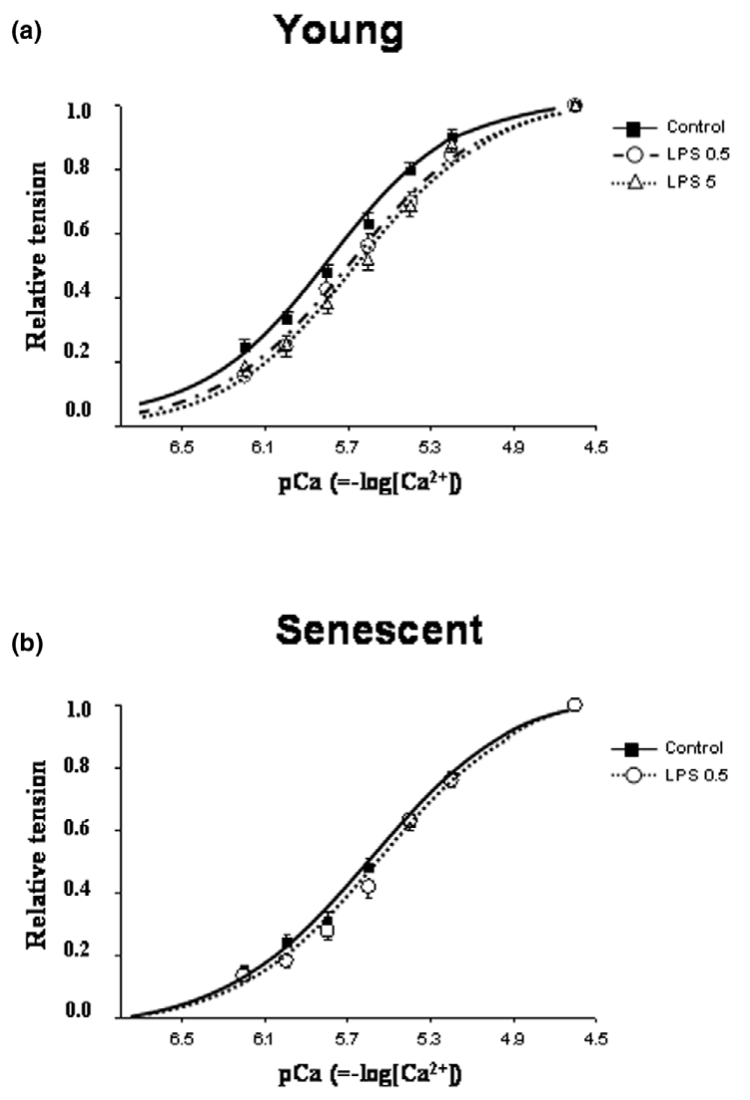
Isometric tension–pCa relations. **(a) **Isometric tension–pCa relations obtained in Triton-skinned left ventricular fibers from young rats (3 months old; control, *n *= 23 fibers; lipopolysaccharide (LPS) 0.5 mg/kg, *n *= 23; and LPS 5 mg/kg, *n *= 22) treated with LPS or saline (control). **(b) **Isometric tension–pCa relations in Triton-skinned left ventricular fibers from senescent rats (24 months old; control, *n *= 22 fibers; LPS 0.5 mg/kg, *n *= 20). Curves of fibers from LPS-treated (0.5 and 5 mg/kg) animals were shifted toward lower pCa values only in young rats, indicating a decrease in Ca^2+ ^sensitivity of the contractile proteins. Values are the mean ± standard error of the mean.

Maximal Ca^2+^-activated tension was similar between endotoxemic animals and their control groups (Table 3). In young rats, LPS 5 mg/kg induced a decrease in myofilament Ca^2+ ^sensitivity (Figure 1a), as attested by a significant decrease in pCa_50 _(0.14 pCa units) without significant change in the Hill coefficient (Table 3). This desensitization was also present in the LPS 0.5 mg/kg group (mean decrease in pCa_50_, 0.12 pCa units; Figure 1a and Table 3). In contrast, skinned fibers from senescent LPS rats exhibited no significant changes in pCa_50 _or in Hill coefficient values (Table 3 and Figure 1b) as compared with fibers from control senescent rats. This suggested that cardiac myofilament Ca^2+ ^sensitivity was not altered during endotoxemia in senescent rats.

### Inflammatory mediator: nitric oxide

**Figure 2:**
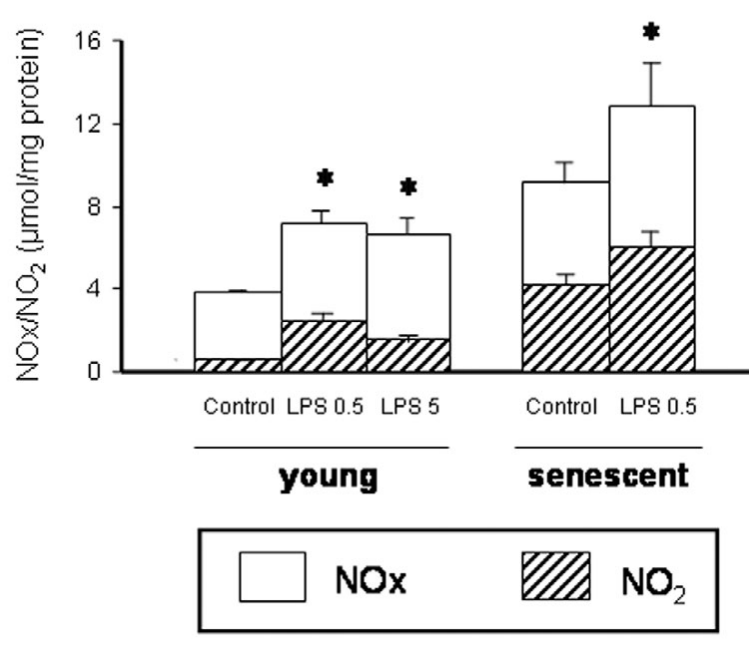
Left ventricular nitric oxide end-oxidation products. Left ventricular nitric oxide end-oxidation products (nitrate + nitrite) (NOx) and NO_2 _contents from young rats (3 months old; control, *n *= 5; LPS 0.5 mg/kg, *n *= 7; LPS 5 mg/kg, *n *= 5) and senescent rats (24 months old; control, *n *= 16; LPS 0.5 mg/kg, *n *= 14) treated with lipopolysaccharide (LPS) or saline (control). Values are the mean ± standard error of the mean. *P < 0.05 versus control group.

Following LPS administration, the NOx and NO_2 _myocardial content increased in a similar fashion in senescent rats and young rats as compared with their respective controls (Figure 2).

### Oxidative stress: TBARS and antioxidant enzyme activities

**Figure 3:**
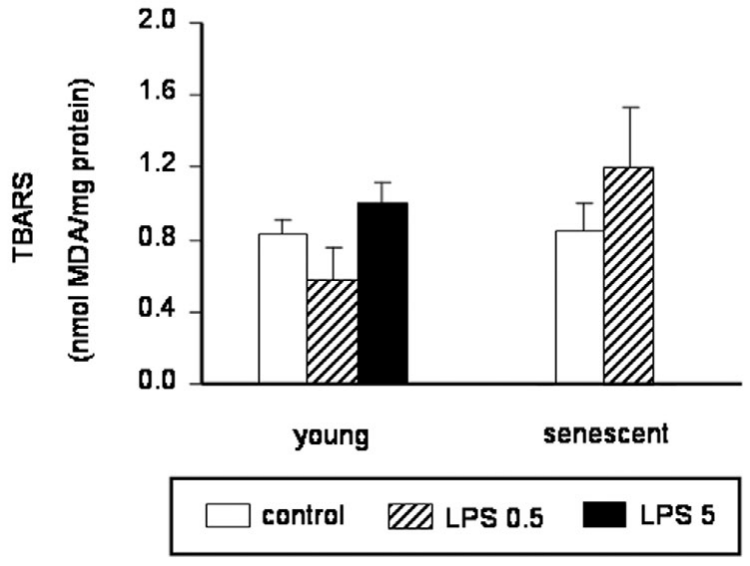
Left ventricular thiobarbituric acid reactive substances content. Left ventricular thiobarbituric acid reactive substances (TBARS) content from young rats (3 months old; control, *n *= 11; lipopolysaccharide (LPS) 0.5 mg/kg, *n *= 8; LPS 5 mg/kg, *n *= 16) and senescent rats (24 months old; control, *n *= 8; LPS 0.5 mg/kg, *n *= 8) treated with LPS or saline (control). Results are expressed as malondialdehyde (MDA) equivalents per milligram of protein (see Materials and methods). The TBARS content was not significantly different in LPS animals versus control animals whatever the age and the LPS dose studied (0.5 or 5 mg/kg in young rats). Values are the mean ± standard error of the mean.

**Figure 4:**
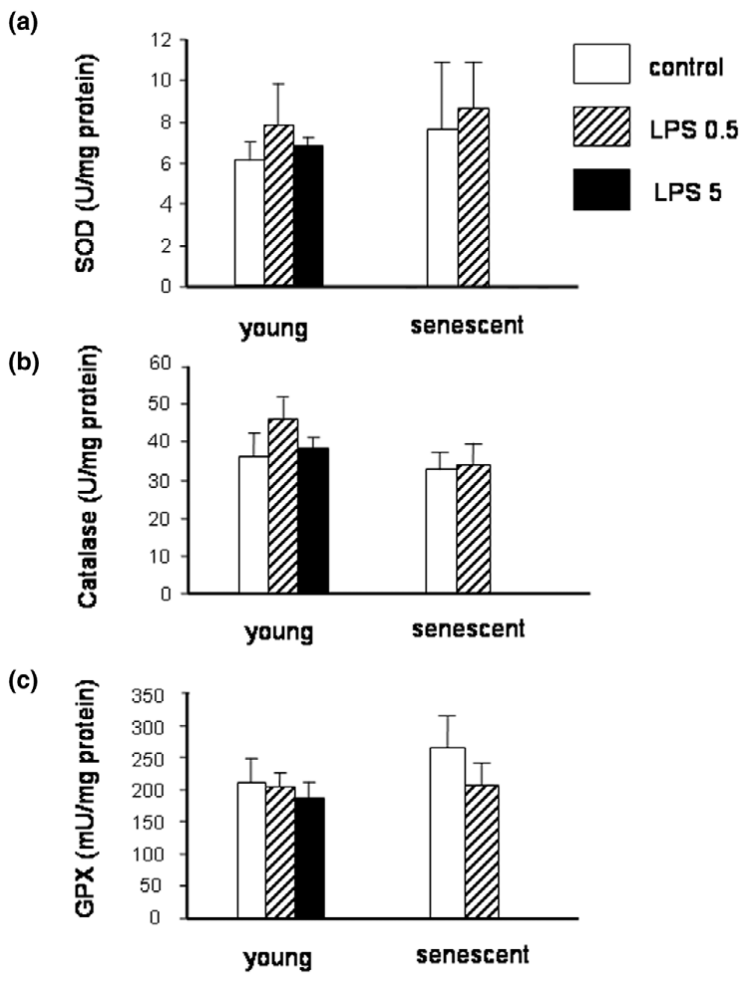
Left ventricular superoxide dismutase activity, catalase activity, and glutathione peroxidase activity. **(a) **Left ventricular superoxide dismutase (SOD) activity in young rats (3 months old; control, *n *= 6; lipopolysaccharide (LPS) 0.5 mg/kg, *n *= 7; LPS 5 mg/kg, *n *= 6) and senescent rats (24 months old; control, *n *= 8; LPS 0.5 mg/kg, *n *= 8) treated with LPS or saline (control). **(b) **Left ventricular catalase activity in young rats (control, *n *= 9; LPS 0.5 mg/kg, *n *= 8; LPS 5 mg/kg, *n *= 11) and senescent rats (control, *n *= 7; LPS 0.5 mg/kg, *n *= 7). **(c) **Left ventricular glutathione peroxidase (GPX) activity in young rats (control, *n *= 9; LPS 0.5 mg/kg, *n *= 8; LPS 5 mg/kg, *n *= 9) and senescent rats (control, *n *= 7; LPS 0.5 mg/kg, *n *= 7). Activities of the three antioxidant enzymes were unchanged during endotoxemia whatever the age and the LPS dose studied (0.5 or 5 mg/kg in young rats). Values are the mean ± standard error of the mean.

Dosages of TBARS in the left ventricle from young rats showed that cardiac lipid peroxidation was not significantly increased in our sublethal model of endotoxemia, whatever the dose of LPS (5 mg/kg or 0.5 mg/kg) administered (Figure 3). Similar results were observed in senescent rats (Figure 3). The CAT, SOD, and GPX myocardial activities were not significantly altered in any LPS group (Figure 4).

## Discussion

The present study tested whether aging alters endotoxin-induced myocardial dysfunction in a sublethal model of endotoxemia in rats. The main findings were the following: 12 hours after LPS injection (0.5 mg/kg), a marked reduction in myocardial contractility was observed in the isolated perfused senescent heart; in contrast with septic cardiac dysfunction in young rats, myofilament Ca^2+ ^sensitivity of left ventricular skinned fibers was not reduced in senescent rats; and NO production, oxidative stress, and antioxidant enzymes activities were not different between young adult and senescent LPS groups. Thus, despite similar alterations in potential mediators, cellular mechanisms responsible for this contractile dysfunction are different between young adult and senescent rats. More specifically, myofilament Ca^2+ ^responsiveness remains unaltered in the senescent heart. This may have clinical implications for management of elderly septic patients.

Nonlethal models of endotoxemia have allowed characterization of septic myocardial depression in young animals while avoiding nonspecific effects of shock [7,12,19]. A reduction by a factor 10 (endotoxin dose from 5 to 0.5 mg/kg) was necessary to reach this objective in senescent rats. This is in accordance with the few other available studies on sepsis in aged animals [5,20,21]. Indeed, mice 24–25 months old have been found to be 10–16 times more sensitive to LPS lethality than young (2 months old) mice [22,23]. The death rate in aged (24 months old) versus young (4 months old) mice has been shown to be dramatically higher after LPS injection or cecal ligation and puncture [21]. This reduction in LPS dose allowed us to compare young rats and senescent rats for cardiac contractile dysfunction of apparently similar severity.

Precise comparison of the amplitude of septic contractile depression between young adult rats and old rats remains speculative, however, as, in accordance with previous studies, indices of contractility in basal conditions were different between the two age groups [2,3]. In our experimental conditions, however, the major contractile depression recorded following LPS in senescent rats versus young rats primarily resulted from reduced performance in basal conditions rather than a larger effect of endotoxemia in old animals. The measurements performed in young rats injected with LPS 0.5 mg/kg show that there is no simple proportional relation between the intensity of mechanical depression, putative mediators such as NO production, and the LPS dose. More importantly, they validate the main results of the study, since they show that the absence of myofilament desensitization in hearts from senescent rats could not be explained by the reduced dose of LPS.

The precise mechanisms for age-dependent vulnerability of the heart to endotoxemia and sepsis remain largely unknown. This can be explained, however, at least in part by reduced physiological reserves and an altered inflammatory response to stress [20,22,24]. The latter may include cardiac NO production. In senescent rats, cardiac constitutive nitric oxide synthase (NOS) activity and cardiac endothelial NOS levels were found higher than in young rats, suggesting that much of this increased NOS activity in the aged heart took place in the endothelium rather than in the myocyte [25,26]. It has also been suggested that the inducible NOS/NO pathway may contribute to ventricular dysfunction during the aging process in mice [27], but these results have not been confirmed in rats [26]. Only one study measured the inducible NOS protein abundance and NOS activity after injection of a cocktail of LPS and IFN-γ in rats, finding that induction of inducible NOS expression and activity was greater in senescent hearts compared with young hearts, while the Ca^2+^-dependent NOS activity was unchanged [26]. Our observation that endotoxemia resulted in an increased NOx accumulation in the myocardium of young rats and of senescent rats is consistent with these findings. Further study should precise the relation between these observations and the depression of contractility in septic senescent animals.

Many studies have suggested that oxidative stress, resulting from mitochondrial dysfunction, excessive reactive oxygen species formation, and altered cellular antioxidant pathways, plays an important role in the development of septic organ dysfunction [28-30]. Following intraperitoneal injection of LPS in rats, the SOD activity and peroxynitrite (3-nitrotyrosine) were elevated in the left ventricle, although they did not parallel the time-course of decreased contractility [28]. In another study using a model of cecal ligature and puncture in rats, mitochondrial dysfunction, an imbalance between SOD and CAT activities, and increased TBARS content have been reported in the myocardium [30]. Moreover, in cells from the senescent heart versus the young heart, an enhanced likelihood for generation of reactive oxygen species during stress as well as an increased susceptibility to undergo protein damage in response to oxidative stress have been reported [5]. Our data in young adult rats as well as senescent rats, however, show that severe septic contractile cardiac dysfunction may occur in the absence of reactive oxygen species-induced cellular alterations in nonlethal endotoxemia. This apparent discrepancy with previous literature can be explained by the results of a recent study showing that oxidative damage, altered antioxidant enzyme activities, and an early increase in TBARS occur in the heart and other tissues essentially during lethal sepsis [31].

Reduced myofilament Ca^2+ ^sensitivity found in left ventricle tissue from young rats in the present study confirms data previously reported in intact cardiomyocytes in rats and in skinned cardiac fibers in rabbits [6,7,32,33]. Myofilament Ca^2+ ^desensitization during sepsis is related to an increase in phosphorylation of troponin I at Ser 23/24 [7]. The difference in pCa_50 _value found between control and LPS fibers in young adult rats is likely to be biologically significant, as we previously showed that the endotoxemia-induced decrease in the pCa_50 _value was as large as that obtained following *ex vivo *incubation of control fibers with isoproterenol (a well established β-adrenergic agonist that induces a protein kinase A (PKA)-dependent decrease in Ca^2+ ^sensitivity of contractile proteins and, in turn, accelerates relaxation in *in vivo *conditions) [9].

Phosphorylation of specific serine and threonine residues on cardiac troponin I by several different protein kinases represents a major physiological mechanism for alteration of myofilament properties [34]. The absence of reduction in myofilament Ca^2+ ^sensitivity found in skinned fibers from senescent hearts thus suggests that protein kinase-dependent phosphorylation of troponin I was attenuated in the septic senescent heart. This difference cannot be attributed to the lower LPS dose used in senescent rats since the same low dose reduced Ca^2+ ^sensitivity in hearts from young rats. A different timescale is also an unlikely explanation as the desensitization has been reported as early as 4 hours after LPS injection and was still present 30 hours later in young endotoxemic animals [6,9,33]. The cAMP-dependent protein kinase (PKA) activity has been found increased in the hearts of young septic animals [35]. This suggests that the PKA pathway may be involved in septic myofilament desensitization. Interestingly, activation of the β-adrenergic–PKA–myofilament protein phosphorylation pathway declines with aging [36]. This may thus contribute to the difference observed in myofilament Ca^2+ ^sensitivity between young hearts and senescent hearts during endotoxemia in the present study. On the other hand, protein phosphorylation via cGMP-dependent protein kinase (protein kinase G), which includes troponin I, seems also decreased during aging [37], thus providing another potential explanation for our observations.

Our study has potential limitations. First, we used a rat model of aging. Rodents differ from humans from a biological point of view, especially concerning body growth. Only a few animal models of aging, however, associate a short lifespan and the control of environmental influences. For cardiovascular studies, the senescent rat is by far the most used animal model of aging. Indeed, physiological changes as well as cellular changes such as myocyte loss, hypertrophy of the remaining myocytes and fibrosis, observed in the human heart during aging, are also found in the senescent rat heart [4,36]. Second, we used three month old rats as controls because at this age septic cardiac dysfunction has been characterized in most previous studies. Three months of age, however, represents young adult rats that are not fully grown and developed. As a result the age difference discussed may not be due to senescence or aging but actually due to maturation. To demonstrate changes due to aging, a mature adult group (aged 9–12 months) would be required. Third, the pertinence of acute endotoxemia as a model for human sepsis has been questioned. The cellular and molecular mechanisms of cardiac dysfunction demonstrated in this model, however, have been generally confirmed in more sophisticated models. Finally, extensive characterization of the various potential mechanisms of septic cardiac dysfunction in the senescent heart needs further investigation. In addition, study of cardiovascular dysfunction in septic senescent animals is needed to appreciate the relevance of the present results.

Our results may have clinical implications. First, reduced myofilament Ca^2+ ^sensitivity has been proposed to constitute the cellular basis of the acute ventricular dilation reported in septic patients [9,38]. Indeed, a reduction in myofilament responsiveness is associated with increased length in single cardiac myocytes and increased ventricular distensibility [34]. The reality of this dilation during septic shock in humans, however, has led to conflicting results [38,39]. Our results in aged animals offer a possible explanation at the cellular level for these divergent observations. Second, our finding that a decrease in myofilament responsiveness to Ca^2+ ^is a determinant of intrinsic myocardial depression in septic shock suggested that Ca^2+^-sensitizing agents may be appropriate treatment to improve heart function in sepsis. Animal studies and, more recently, human studies have begun to test this hypothesis [10,11]. Our results in aged rats raise the possibility that these agents might not be as effective in aged patients as in younger patients.

## Conclusion

Aging is associated with decline in cardiac contractility and altered immune function. Septic myocardial dysfunction may thus be altered with aging in its severity, mediators, and/or main cellular mechanisms. This study demonstrated that a low dose of LPS induced a severe myocardial dysfunction in senescent rats. This was accompanied by an increased myocardial NO production without evidence of oxidative stress, similarly to cardiac dysfunction in young adult rats. Ca^2+ ^myofilament responsiveness, however, which is typically reduced in the myocardium of young septic rats, was unaltered in senescent rats. If these results are confirmed in *in vivo *conditions, they may provide a cellular explanation for the conflicting results regarding the reality of ventricular distensibility during septic shock. In addition, Ca^2+^-sensitizing agents may not be as effective in aged patients as in younger patients.

## Key messages

• Aging is associated with decline in cardiac contractility and altered immune function; in this study, a low dose of LPS induced a severe cardiac contractile depression in senescent rats.

• Endotoxemia-induced myocardial dysfunction in both young rat and senescent rat groups was accompanied by an increased NO production without evidence of oxidative stress.

• Ca^2+ ^myofilament responsiveness, which is typically reduced in the myocardium of young adult septic rats, was unaltered in senescent rats.

• If these results are confirmed in *in vivo *conditions, they may provide a cellular explanation for the divergent reports on ventricular diastolic function in septic shock. In addition, Ca^2+^-sensitizing agents may not be as effective in aged patients as in younger patients.

## Abbreviations

CAT = catalase; GPX = gluthatione peroxidase; IFN = interferon; LPS = lipopolysaccharide; LVDP = left ventricular developed pressure; NO = nitric oxide; NOS = nitric oxide synthase; NOx = NO end-oxidation products (nitrate + nitrite); pCa = log[Ca^2+^]; pCa_50 _= Ca^2+ ^concentration for half-maximal tension, expressed in pCa; PKA = protein kinase A; SOD = superoxide dismutase; TBARS = thiobarbituric acid reactive substances;

## Competing interests

The authors declare that they have no competing interests.

## Authors' contributions

All authors contributed to the elaboration of the protocol. SR carried out *in vivo *observations, isolated and perfused heart experiments, helped with the skinned fiber experiments and biochemical measurements, performed the statistical analysis, participated in analysis and interpretation of data, and drafted the manuscript. SB helped with the acquisition of data, and participated in interpretation of the data and in correction of the manuscript. HB and EJ participated in the acquisition and interpretation of data. AK carried out enzyme activity measurements and participated in analysis of the data. AM was in charge of NOx measurements, and participated in interpretation of data and correction of the manuscript. BR and BV participated in analysis and interpretation of the data, and in correction of the manuscript. BT coordinated the conception and design of the study, helped with *in vivo *observations and performed skinned fiber experiments, participated in analysis and interpretation of data, and helped to draft the manuscript. All authors read and approved the final manuscript.

## Figures and Tables

**Table 1:** Main characteristics of young rats and senescent rats treated with lipopolysaccharide (LPS) or saline

	Young rats (3 months old)			Senescent rats (24 months old)	
	
	Control group (*n *= 9)	LPS 0.5 mg/kg group (*n *= 12)	LPS 5 mg/kg group (*n *= 9)	Control group (*n *= 13)	LPS 0.5 mg/kg group (*n *= 16)
Initial body weight (g)	315 ± 3	331 ± 8	331 ± 5	451 ± 26	459 ± 15
ΔBW (g)	-1 ± 1	-18 ± 2*	-11 ± 3*	-2 ± 2	-16 ± 2*
Liver (g)	12.1 ± 0.8	10.9 ± 0.4	10.1 ± 0.3*	10.9 ± 0.7	9.2 ± 0.4*
Lung (g)	1.30 ± 0.03	1.46 ± 0.04*	1.45 ± 0.03*	4.14 ± 0.47	6.07 ± 0.60*
Heart weight (g)	0.98 ± 0.02	1.09 ± 0.05*	1.10 ± 0.02*	1.34 ± 0.10	1.60 ± 0.10*
Left ventricular weight (g)	0.78 ± 0.02	0.83 ± 0.03	0.85 ± 0.02	1.00 ± 0.07	1.11 ± 0.06

Values are the mean ± standard error of the mean. ΔBW, body weight 12 hours after treatment - initial body weight. *P < 0.05 versus respective control (saline-injected) group.

**Table 2:** Effects of *in vivo *lipopolysaccharide (LPS) on isolated and perfused hearts of young rats and senescent rats

	Young rats (3 months old)			Senescent rats (24 months old)	
	
	Control group (*n *= 8)	LPS 0.5 mg/kg group (*n *= 9)	LPS 5 mg/kg group (*n *= 9)	Control group (*n *= 11)	LPS 0.5 mg/kg group (*n *= 13)
LVDP (mmHg/g HW)	100 ± 7	64 ± 6*	48 ± 7*	53 ± 4	25 ± 4*
dP/d*t*_max _(mmHg/s/g HW)	2744 ± 186	1611 ± 138*	1216 ± 152*	1804 ± 260	893 ± 158*
-d*P*/d*t*_max _(mmHg/s/g HW)	-1863 ± 61	-1320 ± 116*	-1058 ± 103*	-919 ± 91	-480 ± 59*
Coronary flow (ml/minute/g HW)	14 ± 2	14 ± 1	13 ± 2	10 ± 1	12 ± 1

Values are the mean ± standard error of the mean. LVDP, left ventricular developed pressure; d*P*/d*t*_max_, peak of the positive pressure derivative; -d*P*/d*t*_max_, peak of the negative pressure derivative. *P < 0.05 versus respective control (saline-injected) group.

**Table 3:** Effects of *in vivo *lipopolysaccharide (LPS) on left ventricular skinned fibers of young rats and senescent rats

	Young rats (3 months old)			Senescent rats (24 months old)	
	
	Control group (*n *= 4 rats, *n *= 23 fibers)	LPS 0.5 mg/kg group (*n *= 4 rats, *n *= 23 fibers)	LPS 5 mg/kg group (*n *= 5 rats, *n *= 22 fibers)	Control group (*n *= 4 rats, *n *= 22 fibers)	LPS 0.5 mg/kg group (*n *= 5 rats, *n *= 20 fibers)
Resting tension (mN/mm^2^)	3.2 ± 0.5	3.4 ± 0.4	3.7 ± 0.6	6.6 ± 0.5	4.4 ± 0.4*
Maximal active tension (mN/mm^2^)	47.4 ± 3.8	54.4 ± 3.3	51.5 ± 7.6	43.0 ± 3.2	41.8 ± 4.2
pCa_50 _(pCa units)	5.81 ± 0.04	5.69 ± 0.04*	5.67 ± 0.04*	5.58 ± 0.03	5.53 ± 0.03
Hill coefficient	1.60 ± 0.12	1.66 ± 0.11	1.58 ± 0.09	1.43 ± 0.09	1.57 ± 0.10

Values are the mean ± standard error of the mean. pCa_50_, Ca^2+ ^concentration for half-maximal tension, expressed in pCa units (with pCa = -log[Ca^2+^]). *P < 0.05 versus respective control (saline-injected) group.
